# Self-reported beta-lactam allergy, mislabeling and inappropriate antibiotic use: a study from South India

**DOI:** 10.1017/ash.2025.10049

**Published:** 2025-06-30

**Authors:** Krishna Suresh, Vettakkara Kandy Muhammed Niyas, Sabeer Hameed, Parvathy Vijayamohan, Rajalakshmi Ananthanarayanan

**Affiliations:** Department of Infectious Diseases, KIMSHEALTH, Trivandrum, Kerala, India

## Abstract

This study assessed beta-lactam allergy labels in 300 hospitalized adults using validated scores. Over 50% with penicillin and 21% with cephalosporin allergies were classified as low risk. Among those receiving alternative antibiotics due to inappropriate allergy labels, 44% were low-risk. Findings support structured allergy delabeling programs to enhance antimicrobial stewardship.

## Introduction

Beta-lactam antibiotics, particularly penicillins and cephalosporins, are widely used in clinical settings due to their effectiveness, safety, and broad-spectrum activity. Despite their use, up to 10%—15% of hospitalized patients report allergies, most often to penicillin.^1^ However, studies show that over 90% of these reported allergies are inaccurate when formally evaluated.^2^

Incorrectly labeling patients as beta-lactam allergic may have significant clinical consequences. These patients are frequently prescribed alternative antibiotics that are less effective, more toxic, and broader in spectrum, contributing to the development of antimicrobial resistance, *Clostridioides difficile* infections, prolonged hospital stays, and increased mortality.^2–4^

From an antimicrobial stewardship perspective, delabeling inaccurate penicillin and cephalosporin allergies is essential for improving antibiotic selection and patient outcomes.^5^ One widely adopted tool is the PEN-FAST score, which provides point-of-care risk stratification for penicillin allergy. The PEN-FAST tool assigns 1 point for a penicillin allergy within the past 5 years, 2 points for a history of anaphylaxis, angioedema, or severe cutaneous adverse reaction, and 1 point if treatment was required. A score <3 predicts low-risk allergy with a high negative predictive value, allowing eligible patients to undergo a direct oral challenge (DOC) without prior skin testing.^6^ A similar tool, the CEPH-FAST score, was recently developed and validated for cephalosporin allergy. It uses modified PEN-FAST criteria to stratify risk, with a score of less than 3 indicating low risk, enabling safe delabeling of cephalosporin allergies.^7^

The primary objective of this study was to analyze self-reported beta-lactam allergies, stratify their severity, and assess the proportion of patients who were mislabeled as allergic. Additionally, the study examined the appropriateness of antibiotic prescriptions for patients with reported beta-lactam allergies.

## Methodology

This retrospective study was conducted as part of a quality improvement project at KIMSHEALTH, Thiruvananthapuram, from December 1, 2023, to May 31, 2024. Adult patients (≥18 yr) with a documented penicillin or cephalosporin allergy in the electronic medical record (EMR) were included. Patients with incomplete data or who could not be reached by phone were excluded.

Allergy details and prescription data were extracted from the EMR. A structured telephonic questionnaire was used to collect additional information on the reported allergic reaction, including symptoms, timing, duration since the reaction, and treatment received. Risk stratification was performed using the PEN-FAST and CEPH-FAST tools. A score of <3 was classified as low risk, indicating potential eligibility for a DOC, and these cases were considered inappropriately labeled.

All antibiotic prescriptions recorded in the EMR since the date of allergy tagging were analyzed to evaluate how the allergy label influenced prescribing decisions. Prescriptions in which alternative agents were used instead of guideline-recommended beta-lactams due to inaccurate allergy labels were considered inappropriate.

Descriptive statistics were used to summarize allergy risk categories, the proportion of patients potentially mislabeled, and those who received inappropriate antibiotic prescriptions.

## Results

Of 450 patients identified with beta-lactam allergy, 300 were included. Among them, 239 (79.6%) had penicillin allergy, 56 (18.6%) had cephalosporin allergy, and 6 (2%) had both.

Using PEN-FAST and CEPH-FAST, 125 (51.22%) of penicillin-labeled and 13 (20.96%) of cephalosporin-labeled patients were classified as low risk (score <3) (Table 1). These were considered inappropriately labeled and eligible for DOC.

**Table 1.** Risk stratification based on PEN-FAST and CEPH-FAST scores

Score	Penicillin (N=244)	Cephalosporin (N=62)
0 (very low risk of positive allergy test)	47 (19.26%)	3 (4.8%)
1-2 (low risk of positive allergy test)	78 (31.96%)	10 (16.12%)
3 (moderate risk of positive allergy test)	76 (31.14%)	28 (45.16%)
4-5 (high risk of positive allergy test)	43 (17.62%)	21 (33.87%)

Analysis of allergy history revealed that many patients had non-allergic or mild reactions. For penicillin, 37 patients (15.16%) reported non-allergic causes such as gastrointestinal symptoms or family history, and 86 (35.24%) had mild reactions such as maculopapular rashes or could not recall specific reactions. IgE-mediated reactions were reported in 111 patients (45.49%), and 10 (4%) had serious delayed reactions. Among cephalosporin-labeled patients, 23 (37.09%) had mild reactions and 36 (58.06%) had IgE-mediated reactions (Table 2).

**Table 2.** Distribution of reported reactions in penicillin and cephalosporin allergy

Reaction type	Penicillin (N=244)	Cephalosporin (N=62)
Not allergic: • Family history of allergy • GI and other non-allergy symptoms • Remote childhood reaction, later tolerated • Allergy to some other drugs but not beta- lactams	37 (15.16%) 1 (2.7%) 23 (62.16%) 9 (24.32%) 4(10.81%)	2 (3.2%) 0 (0) 1 (50%) 0 1 (50%)
IgE-mediated reactions: • Anaphylaxis • Angioedema • Breathing Difficulties • Laryngeal Edema • Hypotension • Hives/Urticaria	111 (45.49%) 40 (36.06%) 29 (26.12%) 21 (18.91%) 8 (7.2%) 11 (9.9%) 80 (72.07%)	36 (58.06%) 16 (44.44%) 9 (25%) 12 (33.33%) 2 (5.5%) 6 (16.66%) 26 (72.22%)
Mild reactions: • Maculopapular eruption (with or without itching) • EMR lists allergy but patient does not recall having a reaction	86 (35.24%) 38 48	23 (37.09%) 17 6
Serious types of delayed reactions: Stevens-Johnson syndrome (SJS) Drug-induced hepatitis (DIH) Drug rash eosinophilia systemic symptoms (DRESS)	10 (4%) 9 (90%) 1 (10%) 0	1 (1.6%) 0 0 1(100%)

Among 176 patients with a documented indication for antibiotics (penicillin: 137; cephalosporin: 39), 82 (46.6%) received non-beta-lactam agents. Of these, 12 had a PEN-FAST/CEPH-FAST score of 0, 24 had a score of 1–2, 27 had a score of 3, and 19 had a score of 5. Notably, 36 of these patients (44%) were classified as low risk (score <3) and could have potentially received beta-lactams safely. In the low-risk group, alternative antibiotics prescribed included fluoroquinolones in 16 patients (44.4%), carbapenems in 9 (25%), clindamycin in 5 (13.9%), vancomycin in 3 (8.3%), and macrolides in 3 (8.3%).

## Discussion

This study confirms the widespread inaccuracy of beta-lactam allergy labels and highlights missed opportunities for optimized antibiotic use. Over 50% of penicillin and 21% of cephalosporin labels were classified as low risk using PEN-FAST and CEPH-FAST scores. These findings suggest that a substantial proportion of beta-lactam allergy labels may be inaccurate and amenable to delabeling through structured allergy assessment.

Our results are consistent with previous literature. Devchand et al reported successful delabeling in 63.6% of patients with low-risk histories during pharmacist-led ward rounds, reinforcing the feasibility of structured evaluations without specialist referral.^8^ Similarly, Caturano et al reported that 30% of inpatient penicillin allergy labels were removed via history-taking and DOC.^9^ Iuliano et al also showed that only 18.3% of patients with reported beta-lactam allergies had true hypersensitivity following comprehensive evaluation, including skin testing and oral challenges.^3^

Allergy labeling had a direct impact on antibiotic prescribing. Among the 176 patients who required antibiotics, 82 received non-beta-lactams. Of these, 36 patients (44%) were classified as low risk based on PEN-FAST or CEPH-FAST scores and may have safely received beta-lactams. This finding aligns with studies by MacFadden et al and Trubiano et al, which demonstrated that inaccurate allergy labeling leads to increased use of suboptimal therapies and broader-spectrum agents.^4,10^

PEN-FAST and CEPH-FAST proved practical in our setting, effectively identifying patients who could be safely delabeled. These tools, validated by Trubiano et al and Cox et al, offer a reliable bedside approach to clinical risk stratification.^6,7^ Jacobs et al demonstrated that antimicrobial stewardship team-led interventions can significantly improve allergy documentation and reduce inappropriate antibiotic use, aligning well with our approach.^5^ Despite availability, structured allergy evaluations were not routinely used at our center. In response to the findings of this study, our antimicrobial stewardship team introduced a clinical pharmacist-led protocol mandating allergy history-taking and risk assessment.

Nevertheless, this study has limitations. First, allergy histories were collected primarily through telephone interviews, which introduces recall bias and may lack detailed clinical context. Second, we did not perform skin testing or direct oral challenges to confirm allergy status; our conclusions are based solely on validated clinical scoring tools and reported histories.

## Conclusion

Our study confirms the high prevalence of inaccurate beta-lactam allergy labeling, with over 50% of penicillin and 20% of cephalosporin allergy tags potentially incorrect based on validated scores. This mislabeling resulted in the unnecessary use of alternative antibiotics in patients who may have safely tolerated beta-lactams and for whom they were the preferred therapy. Integrating structured allergy assessments into antimicrobial stewardship can promote safer and more appropriate antibiotic use. Implementing delabeling protocols based on clinical history and risk scoring is feasible and impactful, particularly in resource-limited settings where formal allergy testing or access to allergy specialists is not always available.
